# Translational attenuation and retinal degeneration in mice with an active integrated stress response

**DOI:** 10.1038/s41419-018-0513-1

**Published:** 2018-04-30

**Authors:** Christopher R. Starr, Priyamvada M. Pitale, Marina Gorbatyuk

**Affiliations:** Department of Optometry and Vision Science, School of Optometry, University of Alabama at Birmingham, Birmingham, AL USA

## Abstract

An integrated stress response (ISR), identified in several different animal models of inherited retinal degeneration (IRD), is activated following various cellular stresses. The ISR results in the phosphorylation of eIF2α (p-eIF2α) and a consequent halt in protein synthesis. Although generally protective, persistent elevations in p-eIF2α could lead to cell demise. Therefore, we aimed to determine whether ISR activation is associated with diminished translation rates in mice with IRD. Retinal protein extracts from *rd16* mice at different time points were analyzed and the retinal levels of protein synthesis were assessed using the SUnSET method. We found that *rd16* mice experience persistent ISR activation: p-eIF2α, ATF4, and CHOP were significantly upregulated at P15 and P20. In agreement with ISR activation, we found that *rd16* mice experience translational attenuation at P15. Similar to *rd*16, other IRD models, T17M *RHO*, and *rd10* also demonstrated a decline in protein synthesis, correlating with p-eIF2α elevation. We then assessed the role of PERK and eIF2α in translational attenuation in *rd16* using a PERK inhibitor, GSK2606414. We found that while the treatment significantly reduced p-eIF2α, it did not cause a complete recovery in translation. This suggests that eIF2α is not the only or even the primary point of translational control in IRD, and a second node of translational regulation comprising AKT and mTOR should be evaluated. Surprisingly, we found that AKT-mTOR signaling was diminished in *rd16* and *rd10* retinas, suggesting a potential link between AKT-mTOR and translational inhibition. Therefore, for the first time, this study shows translation attenuation in IRD models, and highlights the potential roles of eIF2α kinases and AKT-mTOR signaling that could grant valuable insight into the potential treatments for IRD.

## Introduction

Leber congenital amaurosis (LCA) is a class of inherited retinal dystrophies, in which there is currently no cure and generally diagnosed within the first few months of life^1,2^. In the United States, LCA cases most commonly involve mutations in the gene encoding the centrosomal protein of 290kD (CEP290)^2^. Various retinal degenerative disorders share cellular signaling pathways including the integrated stress response (ISR)^3–5^. In this study, we analyzed ISR signaling in the retinas of *rd16* mice, a model of LCA harboring a 5-exon deletion in *Cep290*. A variety of stress responses including endoplasmic reticulum (ER) stress, amino acid starvation, and viral infection lead to the activation of one or more kinases that phosphorylate the α-subunit of eukaryotic translation initiation factor 2 (eIF2α) at serine 51. eIF2 is responsible for bringing Met-tRNA to the 43S pre-initiation complex. When eIF2α is phosphorylated, eIF2 can no longer participate in translation, and protein synthesis rates go down. Persistent elevations in p-eIF2α have been shown to lead to cell demise^3,6–8^.

Elevated p-eIF2α levels and translational inhibition are becoming a more common finding in neurodegenerative diseases such as Alzheimer’s disease and tauopathies, but the exact role of the ISR in the pathogenesis of these diseases remains unknown. Recently, restoring translation has shown great promise as a potential therapy in neurodegenerative diseases^6,8,9^. In a model of Tau neurodegeneration, decreasing the PERK branch of the unfolded protein response (UPR) restored the levels of important synaptic proteins and rescued neurons^9^. However, the exact role PERK plays in the retina has not been carefully assessed because detecting phosphorylated PERK (p-PERK) in vivo has proven challenging with current methodology. Although p-PERK has not been detected in the retinas of mice with retinal degenration (RD), the activation of the UPR and ISR has been observed^3,10,11^. Interestingly, a recent study showed a 50% decrease in p-eIF2α in P23H rats following treatment with a PERK inhibitor^3^, but the correlation between the ISR and translation has not been explored in RD.

In this study, we sought to determine if different retinopathies could experience metabolic imbalance through the inhibition of protein synthesis over the course of RD. We adapted the surface sensing of translation (SUnSET)^12^ method to measure translation in normal and degenerating retinas. In addition, we treated *rd16* mice with a PERK inhibitor to determine if PERK is the major eIF2α kinase in RD, and to understand whether eIF2α is the main point of translational control within the retinas of mice with RD.

## Results

### The ISR is persistently active in retinas of rd16 mice

**Fig. 1:**
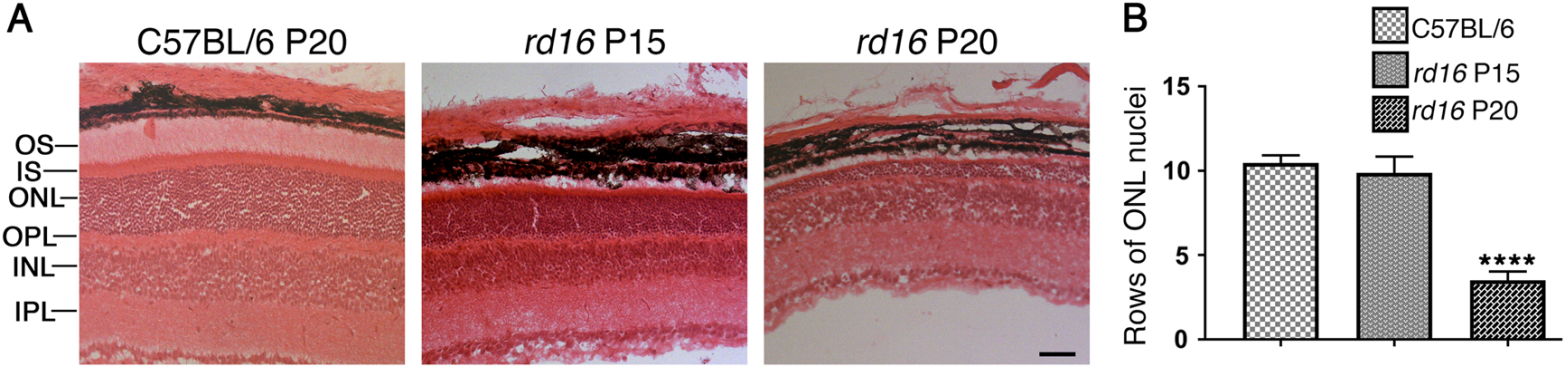
Retinas of *rd16* mice degenerate rapidly. Representative H&E-stained eye sections from C57BL/6 and *rd16* mice (**a**), as well as the corresponding chart displaying the loss of photoreceptor nuclei in the ONL in *rd16* mice at P20 (**b**). OS outer segments, ONL outer nuclear layer (PR nuclei), OPL outer plexiform layer, INL inner nuclear layer, IPL inner plexiform layer. Scale bar represents 100 μm. Data are shown as mean ± SEM. *n* = 3. ****p < 0.0001

**Fig. 2:**
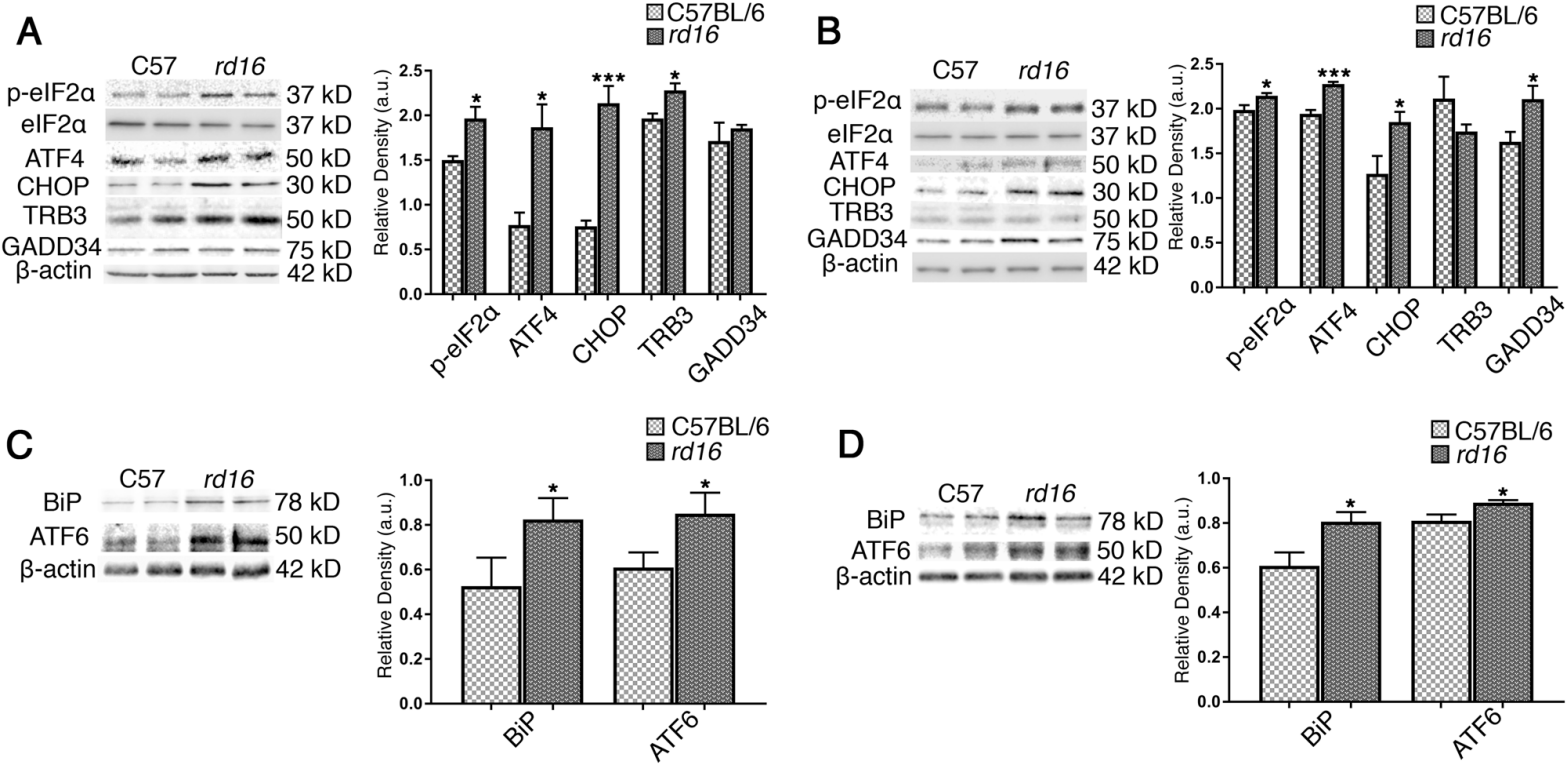
The ISR is active in the retinas of *rd16* mice. Western blots and corresponding graphs of ISR markers in the retinas of *rd16* mice at P15 (**a**) and P20 (**b**). At P15, p-eIF2α, ATF4, CHOP, and TRB3 are significantly elevated. At P20, levels of p-eIF2α, ATF4, CHOP, and GADD34 are increased. Markers of the UPR are elevated in *rd16* retinas. BiP and cleaved (~50 kD) ATF6 are upregulated in the retinas of *rd16* mice at P15 (**c**) and P20 (**d**). Relative density measurements correspond to the intensities of the immunoblotting bands or lanes normalized to an internal control. Data are shown as mean ± SEM. a.u. arbitrary units. P15 *n* = 3. P20 *n* = 4. *p < 0.05, **p < 0.01, ***p < 0.001

Recently, we and other investigators have reported that animal models of varied retinal degenerative disorders share cellular signaling pathways activated over the course of RD^11,13^. T17M *RHO*^14^, P23H *RHO*^15,16^, *rd10*^17^, and TUPL1^18^ are just a few models of RD with elevated levels of p-eIF2α that are thought to contribute to retinal pathogenesis. Before examining ISR signaling within the retinas of *rd16* mice, we first wanted to confirm the rate of photoreceptor (PR) cell loss^1,19,20^. Rows of nuclei within the outer nuclear layer (ONL), which correspond to the nuclei of PRs, were counted in C57BL/6J (wild type; WT) and *rd16* mice on postnatal day (P)15 and P20 (Fig. 1). The number of nuclei did not significantly differ from WT (*n* = 3) at P15; however, PR nuclei were greatly reduced by P20 (*n* = 3). To determine if the ISR is active in the retinas of *rd16* mice, we assessed the levels of ISR markers at two time points, P15 (*n* = 3) and P20 (*n* = 4). Using an antibody specifically against p-eIF2α, we found that it was upregulated at both P15 (Fig. 2a) and P20 (Fig. 2b). We next assessed markers downstream of eIF2α phosphorylation. During the ISR, an alternative translational program is activated and activating transcription factor 4 (ATF4) is selectively translated^21^. ATF4 regulates the expression of several stress response genes including the genes encoding C/EBP homologous protein (CHOP), growth arrest/DNA damage inducible 34 (GADD34), and Tribbles Homolog 3 (TRB3)^21^. Both ATF4 and CHOP were elevated at P15 and P20 (Fig. 2), suggesting that the ISR translational program is active in *rd16* mice. TRB3 was elevated at P15 while GADD34 was elevated at P20.

We next aimed to determine if ER stress may be contributing to the active ISR in the retinas of *rd16* mice. We assessed the levels of two markers of ER stress, binding immunoglobulin protein (BiP) and ATF6. BiP is an ER resident protein chaperone that assists in the folding and clearing of aberrant proteins. Levels of BiP typically go up during ER stress and it is a well-established marker of the UPR. BiP was elevated at both P15 and P20 (Fig. 2c, d) in *rd16* retinas. Aside from the beforehand-mentioned PERK pathway, two additional UPR signaling cascades exist. One of these pathways is signaled through ATF6. Following an ER insult, ATF6 travels to the Golgi body where it is cleaved by sites 1 and 2 proteases. The cleaved ATF6 migrates to the nucleus to act on the genes encoding proteins involved in ER-associated degradation^21^. We assessed levels of cleaved ATF6 (~50 kD) and found that it was elevated at both P15 and P20 (Fig. 2c, d). These results indicate that the UPR may be active in the retinas of *rd16* mice.

### Protein synthesis is inhibited in retinal degeneration

**Fig. 3:**
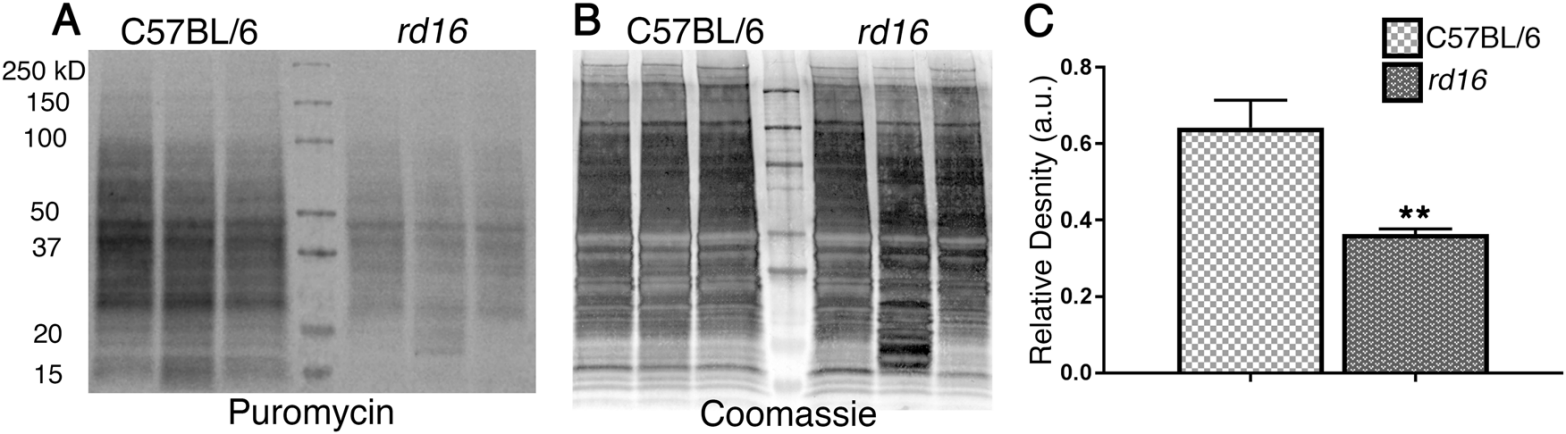
Retinas of mice with inherited RD experience translational inhibition. Measurement of newly synthesized polypeptides using the SUnSET method. Translational attenuation in the retinas of *rd16* mice at P15. Anti-puromycin immunoblotting showing diminished translation in *rd16* mice (**a**). Puromycin immunoblotting was normalized to Coomassie-stained proteins (**b**). Graph comparing levels of translation in *rd16* mice at P15 (**c**). Relative density measurements correspond to intensities of the immunoblotting bands or lanes normalized to an internal control. *n* = 4. Data are shown as mean ± SEM. a.u. arbitrary units. **p < 0.01

Interestingly, various models of RD experience persistent phosphorylation of eIF2α^14,18,21,22^. For example, T17M *RHO* mice, which mimic autosomal-dominant retinitis pigmentosa, demonstrate the elevation of p-eIF2α at P15 and P30^11,14^. *rd10* mice, with a point mutation in PDE6β, model autosomal recessive retinitis pigmentosa (ARRP) and have been shown to experience the activation of ISR signaling in their retinas as early as P15, as shown by an increase in p-eIF2α and CHOP levels^17^. When p-eIF2α is elevated at consequent time points, it may indicate that steady-state translational inhibition is occurring during the course of RD^21^. Therefore, by adopting the SUnSET method^12^, we assessed the levels of newly synthesized proteins in *rd16* at P15 (Fig. 3). This method is based on immunological detection of the incorporation of puromycin (puro), an aminoacyl tRNA-analog, into newly synthesized polypeptide chains. Mice were injected with puromycin at a dose of 0.04 μmol/g body mass 30 min prior to retina isolation. Puromycinylated proteins were separated by SDS-PAGE, transferred to a membrane, and then detected with an antibody against puromycin. By measuring the relative density of a membrane treated with an anti-puro antibody, one can evaluate the rate of protein synthesis. The puro measurements were then normalized by Coomassie staining (Fig. 3b). Figure 3 demonstrates that *rd16* mice experience a ~43% reduction in the rate of protein synthesis at P15. We next wanted to determine if other models of RD could experience translational inhibition. We found that both *rd10* and T17M *RHO* mice have a reduction in translation (Fig S1). Interestingly, *rd10* mice, with a slower rate of RD, demonstrated large translational inhibition (Fig S1A) at P20 (*n* = 4). Meanwhile, T17M *RHO* (Fig S1B) demonstrated a 40% reduction in protein synthesis at P15, as compared to WT retinas (*n* = 4).

### PERK inhibition does not completely restore the translation rates in RD

**Fig. 4:**
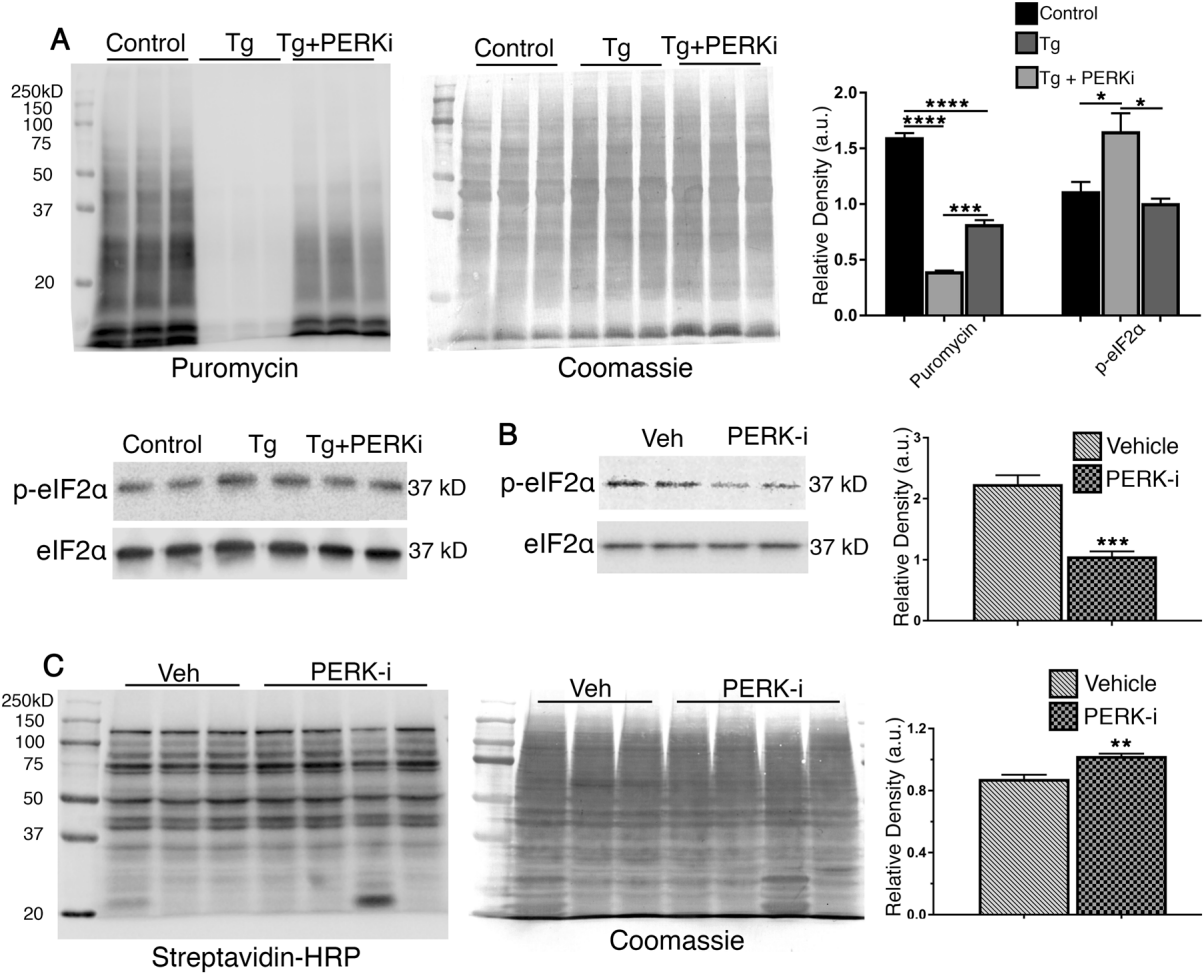
PERK inhibition reduces eIF2α to normal levels, but only partially restores the translation in *rd16* mice and a PR cell line. Treatment with GSK2606414 (PERK-i) brings p-eIF2α down to normal levels, but only partially restores the translation in mouse cone-derived 661 W cells subjected to ER stress (**a**). Cells were treated with 3 μM thapsigargin to induce ER stress (Tg) or DMSO as a control (*n* = 3 plates of cells). Treatment of *rd16* mice (*n* = 3 for vehicle, *n* = 4 for PERK-i) with PERK-i (10 mg/kg) for 2 days brings the retinal levels of p-eIF2α down by ~47% (**b**). Diminished levels of p-eIF2α significantly restores the translation, but only by ~14%. Relative density measurements correspond to intensities of the immunoblotting bands or lanes normalized to an internal control. Data are shown as mean ± SEM. a.u. arbitrary units. *p < 0.05, **p < 0.01, ***p < 0.001, ****P < 0.0001

Four kinases are known to inhibit translation by acting on eIF2α in a variety of conditions^23^. Determining exactly which kinases are involved can be challenging due to various factors. Studies have shown that multiple kinases can become active in certain conditions^23^. Therefore, we first aimed to determine if PERK could play a role in translational attenuation in RD, utilizing a cell culture approach. We inhibited PERK in mouse cone-derived 661W  cells and assessed its role in the translational inhibition following the induction of ER stress with thapsigargin (Tg), a SERCA channel inhibitor. Cells were pre-treated with a PERK inhibitor (PERK-i), GSK2606414, at a concentration of 3 μM for 30 min before the addition of Tg (3 μM) for 2 h. For the last 30 min of Tg treatment, cells were treated with puromycin at a concentration of 10 μM. We found that following PERK inactivation, p-eIF2α was reduced by ~38%, and protein synthesis was significantly restored (Fig. 4a); however, translation did not return to normal levels, meaning that PERK signaling is not the only factor contributing to diminishing protein synthesis rates, even in a model as specific as ER-stressed cells. We then tested this idea in vivo. To that end, we designed a short experiment to determine how much PERK and eIF2α contribute to the translational attenuation in *rd16* mice. Mice were treated with PERK-i (50 mg/kg) by oral gavage twice a day for 2 days. PERK-i has been shown to have excellent central nervous system penetration^9^. Two hours prior to retina collection, mice were injected intraperitoneally with azidohomoalanine (AHA) at a dose of 1.20 mg/kg. AHA is an amino acid analog modified with an azide moiety. Cells take up AHA and incorporate it into the growing peptide chains. In the presence of copper (I), azides react with the conjugated alkyne resulting in tagged proteins^24^. Our AHA-tagged proteins were linked to biotin and separated by SDS-PAGE. The membranes were probed with HRP-conjugated streptavidin and normalized to total protein, as detected by Coomassie staining. We found that the retinas of PERK-i-treated *rd16* mice had a significant decrease (48%) in p-eIF2α (Fig. 4b), suggesting that PERK plays a role in eIF2α regulation in the retina during RD. This was in agreement with a recent study showing that PERK participates in eIF2α phosphorylation in the retinas of P23H rats, as the treatment with PERK-i caused a ~50% reduction in p-eIF2α levels^3^. We next aimed to determine what impact the decline in p-eIF2α had on translation rates. We found that the decrease in phosphorylated eIF2α corresponded to a slight (~16%) but significant increase in the translation rates (Fig. 4c). Altogether, this suggests that eIF2α is not the only, or perhaps even the primary, point of translational control contributing to the decline in the protein synthesis rates in *rd16*, and specific kinase inactivation may not be enough to restore the translation in complex biological systems such as a degenerating retina.

### The AKT-mTOR pathway signals for the decline of translation in mouse models of retinal degeneration

**Fig. 5:**
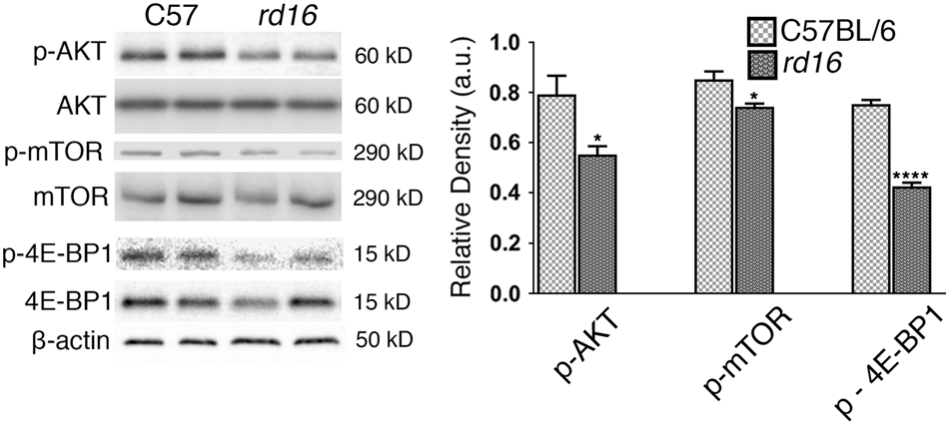
A second node of translational regulation is active in RD. Diminished AKT-mTOR-4E-BP1 signaling in *rd16* mice at P15. *n* = 4. Data are shown as mean ± SEM. a.u. arbitrary units. *p < 0.05, **p < 0.01, ***p < 0.001, ****p < 0.0001

Since the decrease in p-eIF2α levels did not completely restore translation in the retinas of PERK-i-treated *rd16* mice, we next explored a second mechanism of translational control. A second mode of translational control occurs when mTOR signaling is altered^25^. To determine if this second mode of translational inhibition was active in *rd16* mice, we assessed specific markers of the mTOR signaling pathway by western blot at P15 (*n* = 3) (Fig. 5), a time point where all PR nuclei remain. When nutrients are readily available, signaling cascades activate mTOR. However, we found that p-mTOR (Ser2448) was downregulated in the retinas of *rd16* mice at P15. The activation of mTOR results in phosphorylation of eIF4E-binding protein 1 (4E-BP1) and therefore, translational stimulation. When hypophosphorylated, 4E-BP1 interacts with eIF4E, preventing its association into the eIF4F 5′-cap binding complex, thus inhibiting translation^25^. Using an antibody against p-4E-BP1 (Thr37/46), we found that 4E-BP1 phosphorylation was significantly reduced in the retinas of *rd16* mice at P15 (Fig. 5), suggesting that 4E-BP1 is able to interact with eIF4E and inhibit translation initiation. Upstream of mTOR are various factors that regulate its activity including AKT. AKT targets TSC2, preventing it from inhibiting mTOR^25^. We found that p-AKT (Ser473) was downregulated at P15, which is consistent with mTOR inactivation. These data were also in agreement with TRB3 being upregulated at P15 (Fig. 2), a downstream target of ATF4 known to inhibit AKT phosphorylation^26^. These results indicate that the second mode of translational inhibition is active in the retinas of *rd16* mice at P15.

We then assessed whether other models of RD could have both of the translation inhibition elements active. To test the findings in a different mouse model of RD, we utilized western blot analysis to check the levels of p-eIF2α, p-mTOR, and p-AKT in *rd10* mice at two time points, P20 and P25 (*n* = 4). Just as in *rd16*, p-eIF2α was upregulated in the retinas of *rd10* mice at both time points (Fig. S2A and B), which is consistent with the previous reports. In addition, both p-AKT and p-mTOR were downregulated at P20 and P25. Moreover, *rd10* mice demonstrate a significant reduction in p-mTOR as early as P15 (Fig. S2C). In addition, in our previous study, we have also demonstrated that a third RD model, T17M *RHO*, experiences downregulation of p-AKT at P30^27^, and that this reduction correlates with an increase in p-eIF2α^11^. Together, these results indicate that similar to *rd16*, T17M *RHO* and *rd10* undergo translational inhibitory signaling through both ISR and AKT-mTOR signaling.

## Discussion

Although a transient elevation in eIF2α phosphorylation is often physiological and overcame by cells, it is well established that a persistent ISR can lead to apoptosis^3,8,28,29^. An activated ISR results in the global reduction of protein synthesis^23^. This study provides a novel mechanism by which retinas expressing mutant or aberrant proteins in PRs could degenerate and highlights potential therapeutic targets that should be validated in future studies. We revealed that degenerating retinas with persistent phosphorylation of eIF2α exhibit translational inhibition. Second, we found that a second mode of translational control associated with AKT-mTOR signaling is weakened in degenerating retinas.

We found that *rd16* mice had an elevation of p-eIF2α, CHOP, and ATF4 at two time points, demonstrating that *rd16* mice have an activated ISR. Our previous study demonstrated that in degenerating retinas, elevated levels of p-eIF2α are accompanied by the acceleration of RD^13^. Indeed, eIF2α phosphorylation in *rd16* retinas was associated with translational attenuation at P15. The other two mouse models used in this study, T17M *RHO* and *rd10*, develop different ocular phenotypes, but also experience an elevation in levels of p-eIF2α. T17M *RHO* mice demonstrate an increase in eIF2α phosphorylation at P15 and P30^11,14^. At P15, there is no PR cell loss in T17M *RHO* retinas;^11^ however, partial mislocalization of rhodopsin is already observed. *rd10* mice also show an elevation in p-eIF2α levels at P20 and P25 (Fig. S2), and another study has shown p-eIF2α elevation as early as P15^17^. As in *rd16* retinas, we found that protein synthesis was attenuated in the retinas of T17M *RHO* and *rd10* mice, suggesting that translational inhibition is a common theme in RD. However, whether inhibition of translation is a defense mechanism for degenerating PRs or whether a decline in protein synthesis could cause PR cell death needs to be addressed in future studies.

Since there are four kinases that can phosphorylate eIF2α in a variety of conditions, pinpointing the causative factor can be challenging. In fact, ER stress-inducing compounds such as Tg have been shown to activate eIF2α kinases, other than PERK^23^. Our results from in vitro experiments using mouse cone-derived 661W  cells and an in vivo study using *rd16* mice demonstrated only partial restorations in translation rates following treatment with PERK-i. This was in agreement with a recent study that investigated the role of PERK in P23H rats^3^. When the rats were treated with PERK-i, eIF2a phosphorylation was reduced by ~50%. This reduction in eIF2α corresponded to a slight increase in cell death, suggesting that eIF2α phosphorylation could be protective in RD. However, the translation rates were not assessed in these rats, and PERK activity have never been directly shown in the retinas of P23H rats. It would have been interesting to observe the translation rates in these rats, especially since we are now showing that PERK inhibition does not completely restore translation in *rd16* mice. Since PERK ablation is toxic to the pancreas^4^, the use of PERK inhibitors may not be the best approach moving forward. Perhaps a study utilizing retinal specific mutations in PERK and eIF2α would better address these questions. However, we showed that eIF2α may not be the primary point of translational regulation in RD.

We used the SUnSET method to estimate the levels of translation in the retinas of mice with RD. The ability of puromycin to incorporate into nascent peptide chains provides investigators with a powerful tool; however, the SUnSET method is not without its limitations. Detecting in vivo translation rates in the retina means that our dosage must meet some basic requirements. One of these requirements is to use the time point when the studied models retain nearly all of their PRs. While *rd16* outer segments do not develop properly^20^, and this could potentially pose a problem when comparing the groups with similar translational rates, large differences (35–47%; Fig. 3 and Fig. S1) are beyond this limitation.

We also found that AKT-mTOR signaling is weakened in mice with RD. The exact contribution of AKT-mTOR signaling on the translation rates in the retinas of mice with RD, and whether there is cross-talk between the ISR and AKT-mTOR signaling in the retina, are topics that need to be evaluated in future studies. It is entirely possible that AKT-mTOR inactivation could occur independently of the ISR, but several links between the two cascades have been outlined in the literature^25^. For example, TRB3 has been shown to inhibit AKT activation^26^, which could potentially explain how *rd16* mice have reduced p-AKT levels accompanied by an elevation in TRB3 levels at P15. GADD34 is another potential culprit, as it has been shown to result in cytoplasmic retention of AKT, markedly reducing AKT phosphorylation^30^. In addition, ATF4 regulates the transcription of *4EBP1*^31^. Elevated levels of 4E-BP1 would result in more inactivated eIF4E, but our total 4E-BP1 levels did not rise in the retinas of *rd16* mice (Fig. 5). Future studies that look at AKT-mTOR signaling in the absence of TRB3 and GADD34 could help determine if one of these proteins is involved in the potential cross-talk between the ISR and AKT-mTOR signaling. Although we found that translational arrest is associated with RD, there is still a question as to whether inhibition of protein synthesis can cause PR cell death. Interestingly, a recent study identified hypomorphic mutations in the gene encoding tRNA nucleotidyl transferase (TRNT1) that causes retinitis pigmentosa^32^. TRNT1 is required for protein synthesis, and it is likely that mutations in the gene encoding this enzyme lead to RP due to sustained translation attenuation.

T17M *RHO* mice express a protein known to misfold; however, whether *rd16* mice express misfolded CEP290 is not known, which leads to an uncertainty as to why the *rd16* mouse model has an activated ISR. Our observation agrees with a previous study that highlighted active PERK signaling in a BBS12 model^33^. That study utilized an shRNA in ex vivo retinal culture to knockdown BBS12, a ciliary transition zone protein, and the resulting phenotype had an activated UPR. Similar to the retinas of *rd16*, the BBS12 model did not have a gene known to yield a product that aggregates in the ER lumen, but instead displayed abberant protein trafficking to the outer segment, suggesting an issue with intraflagellar transport (IFT). How an abnormal IFT could promote ISR activation remains to be investigated. For example, a stress sensor within the connecting cilium or inner segment could signal for a halt in translation. Additional studies aiming to elucidate the mechanisms responsible for the reduction in translation rates are needed to gain a better understanding of the stresses involved in the progression of RD.

Therefore, for the first time, chronic inhibition of translation has been observed in various models of RD, highlighting translational attenuation as a common theme and potential therapeutic target in RD. We found that retinas with elevated levels of p-eIF2α have reduced translation rates. In addition, we discovered that eIF2α is likely not the primary target of translational regulation in mice with RD, as PERK inhibition brought p-eIF2α down to normal levels but only partially restored translation. Finally, we found that a decline in AKT-mTOR signaling may signal for translational attenuation in RD. Future studies highlighting roles of eIF2α kinases, AKT-mTOR signaling and other means of translational attenuation in the progression of RD could grant valuable insight into potential treatments for RD.

## Materials and methods

### Animals

All animal experiments followed a protocol (IACUC#131109793) approved by the University of Alabama at Birmingham Institutional Animal Care and Use Committee (IACUC), and conformed to guidelines set by the Association of Research in Vision Science and Ophthalmology. *Rd16*, *rd10* and C57BL/6J (Wild type; WT) mice were obtained from Jackson Laboratory (Bar Harbor, ME). T17M *RHO*^+/-^ mice were generated, as previously described^14^. Mice were housed in a facility with a 12 h light/dark cycle. Mice had free access to a standard diet and water. Sample sizes ranged from 3 to 5. At the time points specified in the following sections and text body, mice were euthanized by CO_2_ asphyxiation followed by cervical dislocation.

### Histology

Eyes were enucleated at P15 or P20 and fixed in 4% paraformaldehyde overnight at 4 °C. The fixed eyes were washed with PBS and immersed in 30% sucrose for at least an hour. The eyes were embedded in tissue-tek O.C.T. compound (VWR: 25608-930) and kept at −80 °C for 30 min. Eyes were then cut to 12 μM sections using a cryostat tissue sectioning system (Leica CM 1510S; Leica, Buffalo Grove, IL, USA). The sections were stained with hematoxylin and eosin. The nuclei in the outer nuclear layer were then counted by a masked investigator. The groups were compared by a two-sample *t*-test using GraphPad Prism 7 software.

### Western blotting

For western blot analyses, mouse retinas were dissected and homogenized in lysis buffer (150 mM NaCl, 1.0% Triton X-100, 0.5% sodium deoxycholate, 0.1% SDS, and 50 mM Tris pH 8.0). Protein concentrations were estimated by the Bio-Rad protein assay (5000001, Hercules, CA, USA). The protein lysate (40–60 μg) was separated by SDS-PAGE and transferred to a PVDF membrane for immunoblotting. Primary antibodies specific to phospho-eIF2α (p-S51, 3398, rabbit, lot: 6), eIF2α (9722, rabbit, lot: 15), p-AKT (p-S473, 4060, rabbit, lot: 14), ATF4 (11815, rabbit, lot: 4), β-actin (4970, rabbit, lot: 4), p-mTOR (p-S2448, 2971, rabbit, lot: 21), AKT (4691, rabbit, lot: 20), mTOR (2983, rabbit, lot: 14), p-4E-BP1 (p-Thr37/46, 2855P, rabbit, lot: 20), and 4E-BP1 (9644, rabbit, lot: 10) were purchased from Cell Signaling Technologies (Danvers, MA, USA). Antibodies against TRB3 (sc-34211, goat, lot: A0615), CHOP (sc-7351, mouse, lot: I2614), GADD34 (sc-8327, rabbit, lot: I0810), and GRP78 (sc-1050, rabbit, lot:J3015) were purchased from Santa Cruz Biotechnologies (Dallas, TX, USA). ATF6 (NBP1-40256, mouse) and Novus Biologicals (Littleton, CO, USA). Secondary antibodies (WesternSure HRP goat anti-mouse IgG: 926-80010, WesternSure HRP goat anti-rabbit IgG: 926-80011, IRDye 800CW Donkey anti-goat: 926-32214 and IRDye 800CW Goat anti-mouse: 925-32210) were purchased from LI-COR (Lincoln, NE, USA). The probed membranes were imaged on a LICOR Fc imaging system. Relative band densities were measured using ImageJ software. Westerns were normalized to β-actin. Phosphorylated proteins were normalized to their non-phosphorylated counterparts. The groups were compared by a two-sample *t*-test using GraphPad Prism 7 software.

### in vivo SUnSET

For *in vivo* analysis of nascent peptide synthesis as detected by western blot – T17M *RHO* P15, *rd16* P15, *rd10* P20, and age matched wild type (C57BL/6) mice – were intraperitoneally injected with puromycin (puromycin dihydrochloride; Santa Cruz Biotechnology, CAS 58-58-2) at a dosage of 0.04 μmol/g body mass. After 30 min of the puromycin injection, the retinas were harvested. Proteins were extracted in RIPA buffer, and the proteins were estimated, as stated above in the western blotting section. Between 60 and 80 μg of protein were separated by SDS-PAGE and transferred to a PVDF membrane. PVDF membranes were probed with an anti-puromycin (MABE343, mouse, lot: 2861354) antibody purchased from EMD Millipore. A secondary antibody specific for IgG2a (goat anti-mouse peroxidase affinipure IgG, Fcγ Subclass 2a Specific: 115-035-206, Jackson Immuno Research Laboratories Inc; West Grove, PA, USA) was used to prevent the detection of the heavy and light chains of the endogenous immunoglobulin. As a loading control, the membranes were stained with coomassie blue R-250 (Bio-Rad: #1610436). After anti-puromycin immunoblotting, the membranes were washed with distilled water and then incubated in coomassie staining solution for 60 s. Membranes were then de-stained (ethanol/acetic/water; 5:1:4 proportion) for 10–15 min and air dried before scanning on a Kyocera Taskalfa copier. The relative densities of entire lanes were measured using ImageJ software. Puromycin densities were normalized to their corresponding coomassie densities. Groups were compared by a two-sample *t*-test using GraphPad Prism 7 software. For the PERK-i experiment, *rd16* mice were fed GSK2606414 (Millipore, 516535; Burlington, MA, USA) by oral gavage (in vehicle: 0.5% HPMC, 0.1% Tween-80 in H_2_O at pH 4.0) at a dose of 50 mg/kg twice a day for 2 days. The treatment started when the mice were P14. Two hours prior to retinal collection, the mice were intraperitoneally injected with Click-it Azidohomoalanine (ThermoFisher, C10102; Waltham, MA, USA) at a dosage of 1.20 mg/kg. The retinas were collected and 100 µg of protein were subjected to a click-it reaction, following the protocol outlined by the manufacturer (ThermoFisher, C10276). The reaction involved chemically linking AHA to an alkyne (Biotin Alkyne, B10185). After the tagged proteins were precipitated to remove the reaction components, they were re-solubilized in 1× SDS-loading buffer (2% SDS, 10% glycerol, 0.005% bromophenol blue and 5% 2-mercaptoethanol) and heated at 95 °C for 10 min before loading on a gel and separating by SDS-PAGE.

### Immunocytochemical SUnSET

Mouse cone-derived 661 W cells were plated at a density of 1.78 × 10^5^ cells/cm^2^ and grown to confluency at 37 °C in Dulbecco’s modified eagle medium (DMEM; GE healthcare, SH30022; Chicago, IL, USA), supplemented with 10% fetal bovine serum (FBS) as well as penicillin and streptomycin. Cells were then pre-treated with DMEM + 10% FBS containing either 3 μM PERK inhibitor (GSK2606414) or solvent (DMSO) for 30 min. Thapsigargin (at a final concentration of 3 μM), or DMSO was then added to the media for 2 h. For the last 30 min of treatment, puromycin was added at a final concentration of 10 μM. Proteins were extracted using 1× SDS-loading buffer (2% SDS, 10% glycerol, 0.005% bromophenol blue and 5% 2-mercaptoethanol) and heated at 95 °C for 10 min before loading on a gel and separating by SDS-PAGE. The relative densities of entire lanes were measured using ImageJ software. Puromycin densities were normalized to their corresponding coomassie densities. Groups were analyzed by one-way ANOVA using GraphPad Prism 7 software.

## Electronic supplementary material

Supplemental Fig. 1

Supplemental Fig. 2

Supplemental Figure Legends
