# Supplementary figures and images for: Translational attenuation and retinal degeneration in mice with an active integrated stress response
Source: Cell Death Dis. 2018 Apr 30;9(5):484. doi: 10.1038/s41419-018-0513-1 (PMC5924758; doi:10.1038/s41419-018-0513-1)

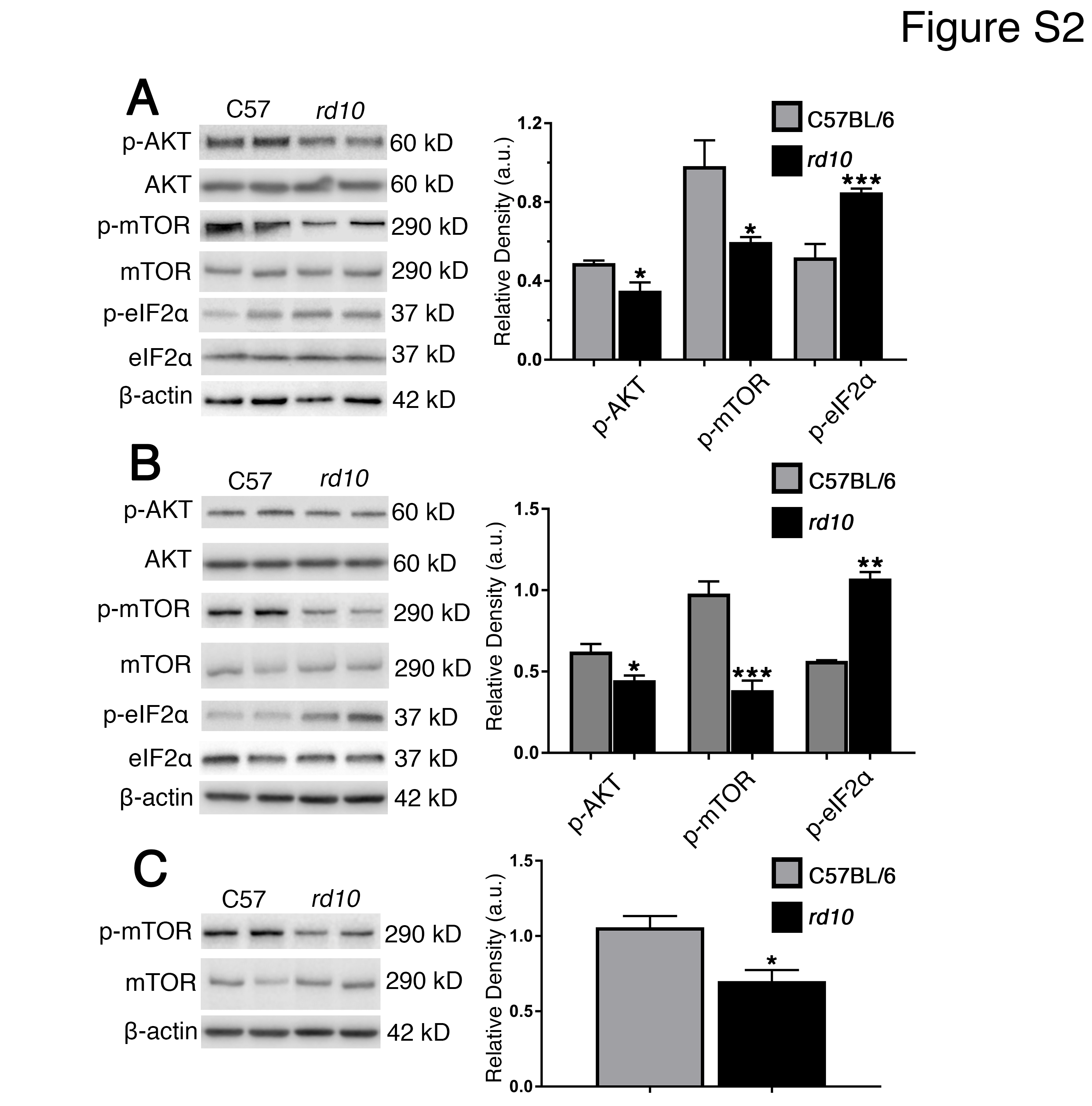

Supplement: Supplementary file 2 — Supplemental Fig. 2 [file 41419_2018_513_MOESM2_ESM.tif]
