# Supplementary material for: Translational attenuation and retinal degeneration in mice with an active integrated stress response
Source: Cell Death Dis. 2018 Apr 30;9(5):484. doi: 10.1038/s41419-018-0513-1 (PMC5924758; doi:10.1038/s41419-018-0513-1)
Supplement: Supplementary file 3 — Supplemental Figure Legends [file 41419_2018_513_MOESM3_ESM.pdf]

Fig S1. Protein synthesis is inhibited in mouse models of RD. Retinas of *rd10* mice experience translational attenuation at P20 (A) (n=4). T17M *RHO* mice display reduced translation at P15 (B) (n=4). Puromycin immunoblotting normalized to total protein as detected by coomassie staining. Data is represented as mean ± SEM. a.u. - arbitrary units. ** = $p < 0.01$.

Fig S2. The retinas of *rd10* mice signal for a halt in translation through both eIF2α and AKT/mTOR. Markers of translational regulation in *rd10* mice at P20 (A) (n=4) and P25 (B) (C57BL/6J : n=5, *rd10* : n=4). The retinas of *rd10* mice have diminished AKT/mTOR signaling and elevated eIF2α phosphorylation. p-mTOR was also reduced at P15 (C) (n=4). Data is represented as mean ± SEM. a.u.- arbitrary units. * = $p <0.05$, ** = $p <0.01$, *** = $p < 0.001$,
